# Comparative Phylogenomic Study of Malaxidinae (Orchidaceae) Sheds Light on Plastome Evolution and Gene Divergence

**DOI:** 10.3390/ijms252011181

**Published:** 2024-10-17

**Authors:** Meng-Yao Zeng, Ming-He Li, Siren Lan, Wei-Lun Yin, Zhong-Jian Liu

**Affiliations:** 1 Key Laboratory of National Forestry and Grassland Administration for Orchid Conservation and Utilization at College of Landscape Architecture and Art, Fujian Agriculture and Forestry University, Fuzhou 350002, China; 62419052001@fafu.edu.cn (M.-Y.Z.); fjalmh@fafu.edu.cn (M.-H.L.); lkzx@fafu.edu.cn (S.L.); 2 Fujian Colleges and Universities Engineering Research Institute of Conservation and Utilization of Natural Bioresources, Fujian Agriculture and Forestry University, Fuzhou 350002, China; 3 College of Biological Sciences and Technology, Beijing Forestry University, Beijing 100083, China

**Keywords:** Malaxidinae, plastid genome, comparative genomics, phylogeny

## Abstract

Malaxidinae is one of the most confusing groups in the Orchidaceae classification. Previous phylogenetic analyses have revealed that the relationships between the taxa in Malaxidinae have not yet been reliably established, using only a few plastome regions and nuclear ribosomal internal transcribed spacer (nrITS). In the present study, the complete plastomes of *Oberonia integerrima* and *Crepidium purpureum* were assembled using high-throughput sequencing. Combined with publicly available complete plastome data, this resulted in a dataset of 19 plastomes, including 17 species of Malaxidinae. The plastome features and phylogenetic relationships were compared and analyzed. The results showed the following: (1) Malaxidinae species plastomes possess the quadripartite structure of typical angiosperms, with sizes ranging from 142,996 to 158,787 bp and encoding from 125 to 133 genes. The *ndh* genes were lost or pseudogenized to varying degrees in six species. An unusual inversion was detected in the large single-copy region (LSC) of *Oberonioides microtatantha*. (2) Eight regions, including *ycf1*, *matK*, *rps16*, *rpl32*, *ccsA*-*ndhD*, *clpP*-*psbB*, *trnF^GAA^*-*ndhJ*, and *trnS^GCU^*-*trnG^UCC^*, were identified as mutational hotspots. (3) Based on complete plastomes, 68 protein-coding genes, and 51 intergenic regions, respectively, our phylogenetic analyses revealed the genus-level relationships in this subtribe with strong support. The *Liparis* was supported as non-monophyletic.

## 1. Introduction

The Malaxidinae subtribe (Orchidaceae, Malaxideae) consists of approximately 1250 species in 14 genera and is sister to Dendrobiinae [1,2,3]. Malaxidinae species are terrestrial; occasionally epiphytic; rarely holomycotrophic; and characterized by fleshy stems, terminal and racemose inflorescences, and small flowers [4]. This subtribe is primarily distributed in the tropics and subtropics, with only a few genera extending into temperate regions (*Liparis*, *Malaxis*, and *Oberonia*) [3]. Malaxidinae possesses remarkable ornamental value owing to its distinctive flowers. Additionally, this subtribe holds importance in traditional Chinese medicine, plant chemistry, and pharmacology [5,6].

Malaxidinae species show limited morphological variations, leading to unclear taxonomic boundaries at the species and genus levels, making them a challenging group for taxonomic research [4,7,8,9,10]. Previous studies based on traditional molecular markers have consistently shown that the phylogenetic relationships of Malaxidinae have moderate to low support values and highly unstable topological structures. Cameron et al. [4] proposed dividing Malaxidinae into at least seven genera using the nuclear ITS and plastid *matK* regions. Phylogenetic analysis has also revealed that *Oberonia* was monophyletic, while *Liparis* s.l. (including terrestrial and epiphytic) and *Malaxis* s.l. were polyphyletic. Based on four markers, ITS, *matK*, *trnS*-*trnG*, and *trnL*-*trnF*, Tsutsumi et al. [11] showed that *Liparis* was polyphyletic, as *Malaxis* species were nested within *Liparis*. Other phylogenetic analyses of Malaxideae have mainly employed ITS and *matK*, with extended sampling including *Crepidium*, *Crossoglossa*, *Crossoliparis*, *Dienia*, *Hippeophyllum*, *Stichorkis*, and *Tamayorkis* [2,12,13,14,15,16,17]. Despite the expansion of genus sampling in these studies, the issue of non-monophyly in *Liparis*, *Malaxis*, and *Crepidium*—as well as low support values and unstable topological structures—remains unresolved. The phylogenetic relationships of Malaxidinae were still difficult to determine, necessitating alternative approaches to increase resolution.

Plastomes are characterized by a high copy number per cell and a comparatively small size, making complete sequencing fairly simple. Due to a lack of recombination and low nucleotide substitution rates, and usually uniparental inheritance [18,19], plastomes have found widespread applications in addressing issues such as node collapse and have low support values based on traditional molecular markers [20,21,22,23]. A few Malaxidinae plastome sequences, including *Liparis*, *Oberonia*, and *Oberonioides*, have been published in the GenBank database (https://www.ncbi.nlm.nih.gov/ (accessed on 6 May 2022)) [24,25,26,27,28], but detailed analyses and comparisons of their composition and structure have not been conducted. In this study, we present two newly sequenced plastomes of *Oberonia integerrima* Guillaumin and *Crepidium purpureum* (Lindl.) Szlach. and compare them with 17 previously published Malaxidinae plastome sequences (from 15 species). We analyzed differences in genome size, content, structure, the inverted repeat (IR) contraction and expansion, and codon-usage bias and identified sequence divergence and variant hotspot regions. In addition, we reconstructed the phylogenetic trees of Malaxidinae based on plastome datasets. The results provide a useful genetic resource for the molecular identification and evolutionary studies of Malaxidinae.

## 2. Results

### 2.1. Plastome Features and Genome Rearrangement

**Figure 1:**
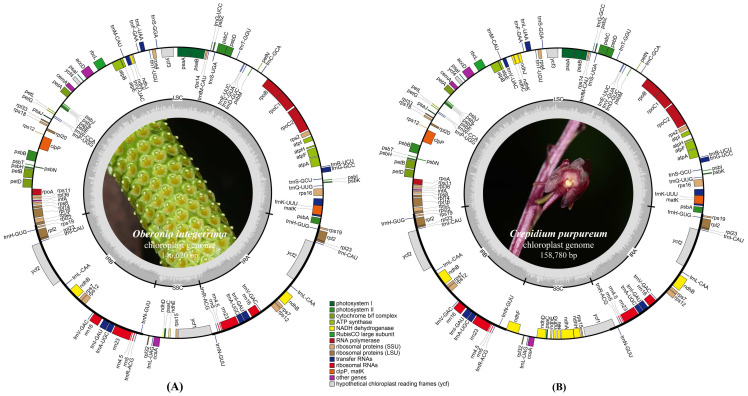
Annotation map of the plastomes for *Oberonia integerrima* (**A**) and *Crepidium purpureum* (**B**).

The *Oberonia integerrima* and *Crepidium purpureum* plastome data were submitted to GenBank (https://www.ncbi.nlm.nih.gov/ (accessed on 11 November 2023)) with accession numbers OR702584 and OR702583. The lengths of the newly sequenced plastomes were 146,620 bp and 158,780 bp, and the GC contents were 36.8% and 37.4%, respectively (Figure 1). In the typical quadripartite structures of these two species, the length and percentage of the large-single copy (LSC) regions were 83,791 bp (57.15%) and 86,383 bp (54.40%), the IR regions were 25,821 bp (17.61%) and 27,015 bp (17.01%), and the small-single copy (SSC) regions were 11,187 bp (7.63%) and 18,367 bp (11.57%), respectively.

In *O. integerrima*, 126 annotated genes and 75 protein-coding genes were detected; *ndhC*, *ndhF*, *ndhG*, *ndhI*, *ndhA*, and *ndhH* were lost and *ndhJ*, *ndhK*, *ndhB*, and *ndhD* were pseudogenized. However, the *ndh* gene was neither lost nor pseudogenized in *C. purpureum*, and 132 annotated genes and 86 protein-coding genes were detected. A total of 38 transfer RNA (tRNA) and eight ribosomal RNAs (rRNAs) were annotated in these two species (Table S1).

In total, 19 Malaxidinae plastomes, including *Liparis*, *Crepidium*, *Oberonioides*, and *Oberonia*, were analyzed in this study. The plastomes ranged from 142,996 bp (*O. japonica* (Maxim.) Makino) to 158,787 bp (*L. nervosa* (Thunb.) Lindl.), with of 36.8% to 37.4% GC content. All plastomes displayed a typical quadripartite structure. The length and percentage of the LSC region ranged from 81,669 bp (57.11%) to 86,752 bp (54.82%), the IR region ranged from 24,003 bp (16.56%) to 27,268 bp (17.17%), and the SSC region ranged from 10,224 bp (7.15%) to 18,367 bp (11.57%).

**Figure 2:**
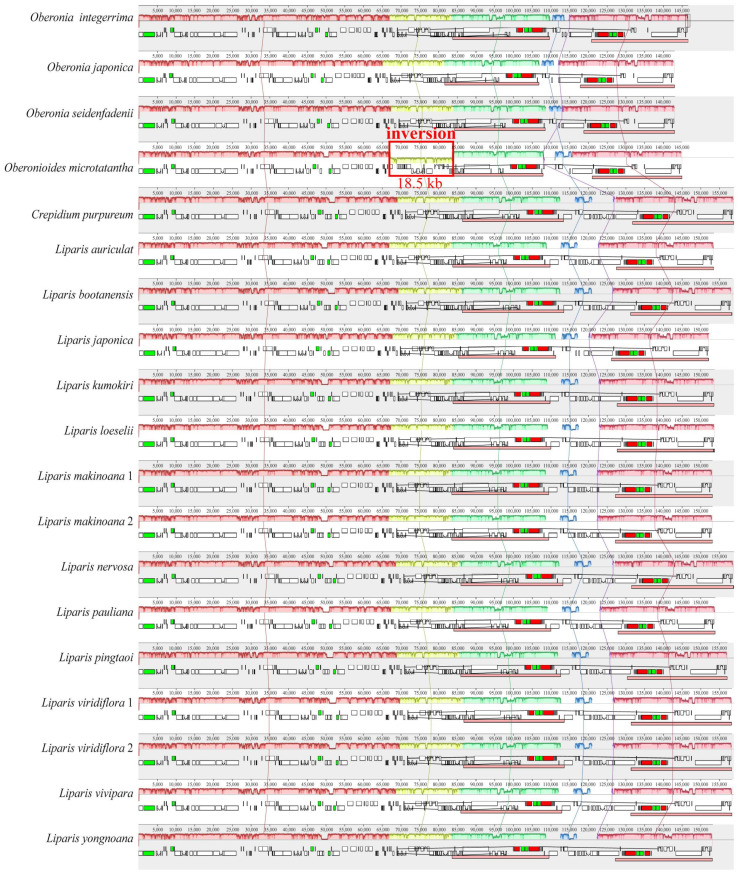
Alignment of 19 Malaxidinae plastomes using Mauve. Comparative gene maps showed an inversion in the *rpl33*-*rps3* region of *Oberonioides microtatantha*. The locally collinear blocks are represented by blocks of the same color connected by lines. Genome regions are color-coded as CDS, tRNA, rRNA, and non-coding region.

The annotated genomes encoded 125–132 genes, of which *O. japonica* and *O. seidenfadenii* (H.J.Su) Ormerod had the least genes, containing 74–86 protein-coding genes, 38 tRNA genes, and 8 rRNA genes (Table S1). The *ndh* gene was lost or pseudogenized to varying degrees in six species, including *Oberonia integerrima*, *O. japonica*, *O. seidenfadenii*, *Oberonioides microtatantha* (Tang & F.T.Wang) Szlach., *L. japonica* (Miq.) Maxim., and *L. yongnoana* N.S.Lee, C.S.Lee & K.S.Lee. Furthermore, the plastome rearrangements were analyzed using Mauve. An inversion of approximately 18.5 kb in the *rpl33*-*rps3* region was identified in the LSC region of *Oberonioides microtatantha* (Figure 2).

### 2.2. IR Region Border Analysis

**Figure 3:**
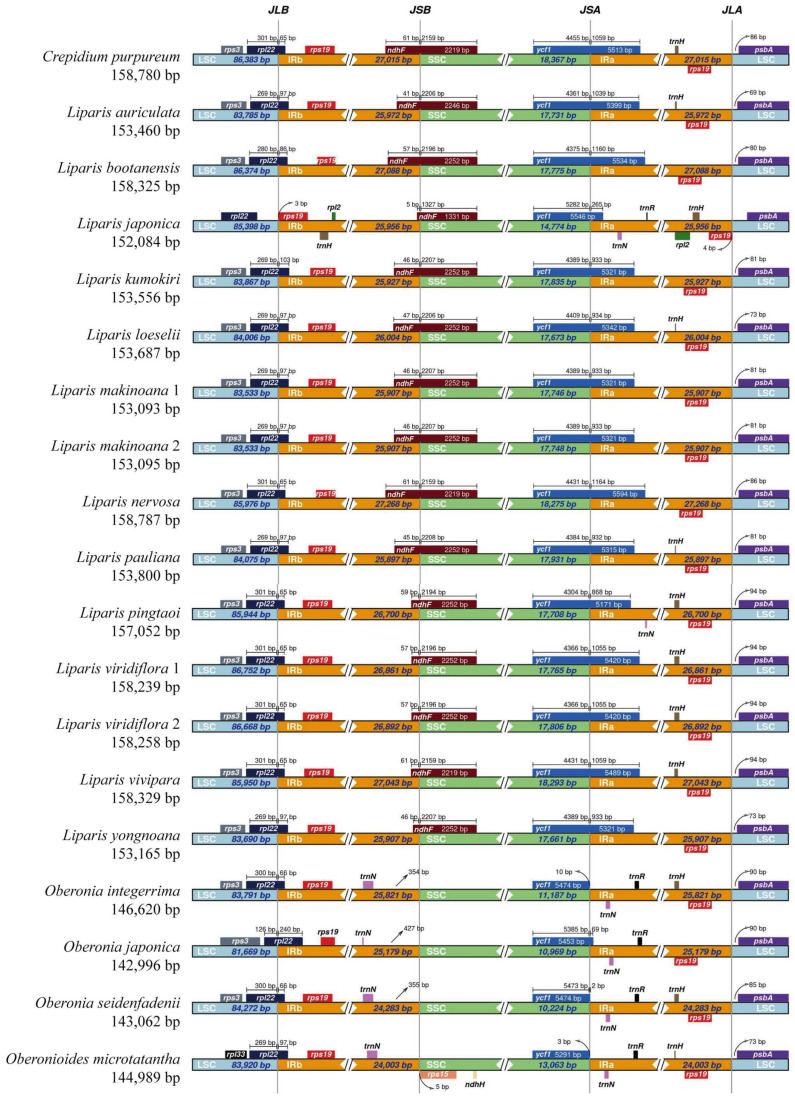
Comparison of boundaries between the LSC, SSC, and IR regions among 19 Malaxidinae plastomes.

The IR/SC region border in Malaxidinae plastomes showed a relatively conserved structure (Figure 3). An except for the *rpl22* gene of *L. japonica* was located in the LSC region, and the *rpl22* genes of other species spanned the IRb/LSC (JLB) boundary. The IRb/SSC (JSB) boundary was located in *ndhF*; except for four species of *Oberonia* and *Oberonioides*, the JSB boundary was located between the *trnN^GUU^* and *rps15* genes. The *ycf1* gene was located in the SSC region in *Oberonioides microtatantha* and *O. integerrima*, but in other species it spanned the IRa/SSC (JSA) junction. The IRa/LSC (JLA) border of all species was located between *rps19* and *psbA*.

### 2.3. Codon Usage Analysis

**Figure 4:**
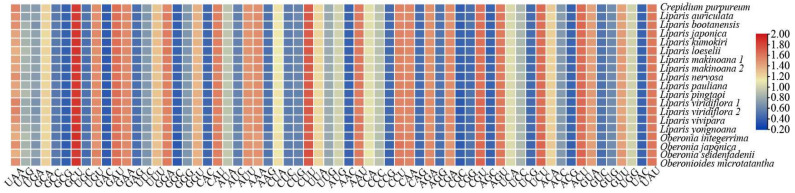
The RSCU values of 68 concatenated protein-coding genes for Malaxidinae plastomes. Color key: the red values indicate higher RSCU values and the blue values indicate lower RSCU values.

After extracting the protein-coding genes and removing the repeated genes, the matrices of 68 unique coding genes were obtained. The 68 protein-coding genes encoded 19,247–19,464 codons among the 19 plastomes (Table S2). The most frequent amino acid was leucine (Leu), while the least frequent was cysteine (Cys). Additionally, the synonymous codon usage (RSCU) values were calculated, ranging from 0.360 to 1.899, and a heatmap revealed that codon frequencies were largely similar within Malaxidinae. The GCU codon had the highest RSCU value (1.844–1.899), while GAC exhibited the lowest (0.360–0.381) (Figure 4, Table S2). Among three termination codons (UAA, UAG, and UGA) the RSCU value of the UAA codon (1.368–1.544) was the highest (Table S2).

### 2.4. Nucleotide Mutation Hotspots

**Figure 5:**
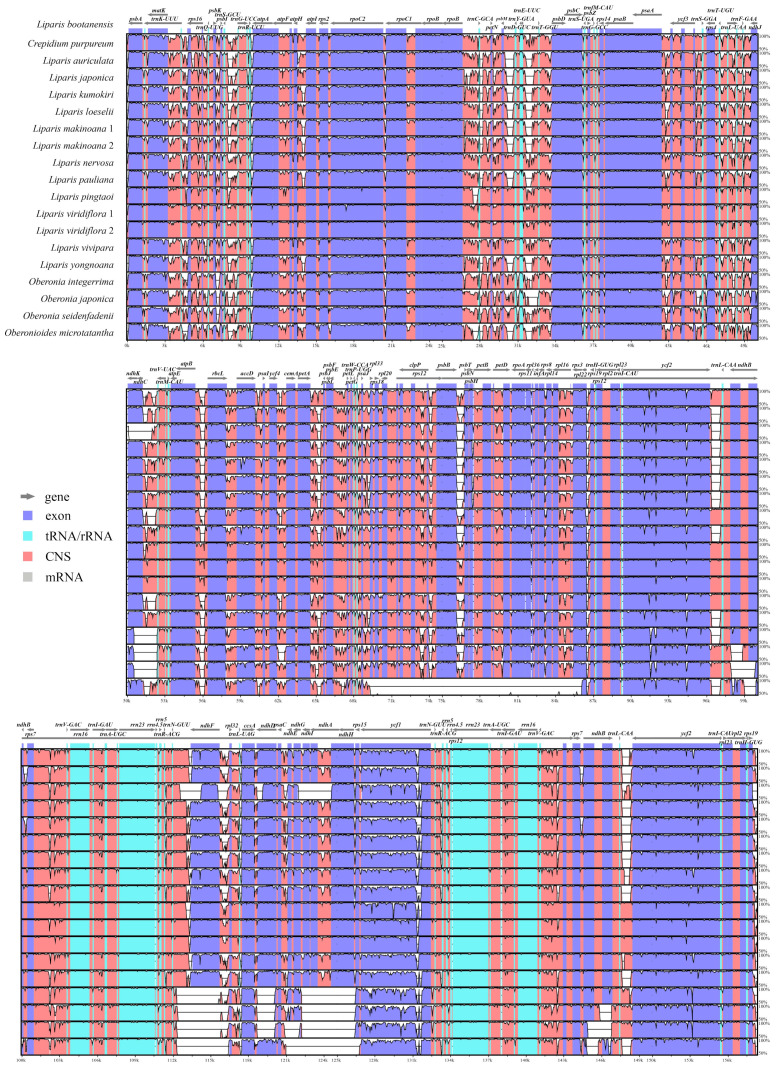
mVISTA map of Malaxidinae plastomes with *L. bootanensis* as reference. The y-axis shows the coordinates between the plastomes.

**Figure 6:**
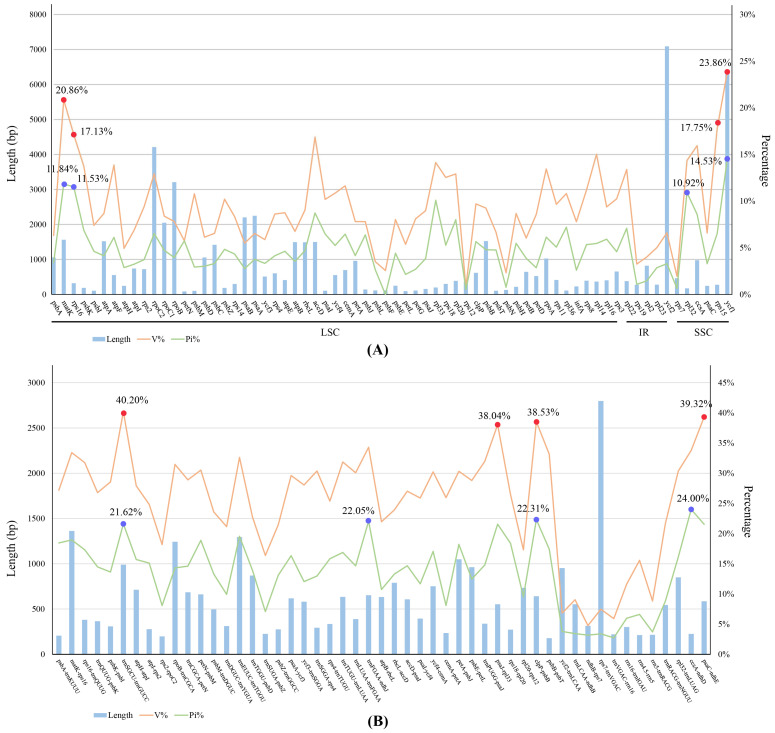
Nucleotide mutation hotspots of protein-coding region (**A**) and intergenic region (**B**) of Malaxidinae plastomes. The red and blue points indicate the top four proportion of the variable sites and parsimony information sites, respectively, with the protein-coding region and the intergenic region.

The sequence identity was compared and plotted using the mVISTA program. The results showed that the protein-coding genes were more highly conserved than the intergenic regions (Figure 5). The IR regions were more conserved than the LSC and SSC regions. The 68 unique protein-coding genes were 69,810 bp long; variable sites (V) were 7153 (accounting for 10.24% of the total length) and the parsimony informative sites (Pi) were 3798 (5.44%). Among the individual protein-coding gene, the variable site values ranged from 0.81% (*rps12*) to 23.86% (*ycf1*) and the Pi values ranged from 0% (*psbF*) to 14.53% (*ycf1*) (Figure 6, Table S3), and the *ycf1*, *matK*, *rps16*, and *rpl32* genes exhibited higher Pi values (14.53%, 11.84%, 11.53%, and 10.92%). Regarding 51 unique intergenic regions, the combined dataset was 30,003 bp long, of which the variable sites were 7694 (25.64%) and the Pi were 4137 (13.79%) (Figure 6, Table S3). The Pi values ranged from 2.54% (*rps7*-*ndhB*) to 24.00% (*ccsA*-*ndhD*); notably, the *ccsA*-*ndhD*, *clpP*-*psbB*, *trnF^GAA^*-*ndhJ*, and *trnS^GCU^*-*trnG^UCC^* regions had higher Pi values (24.00%, 22.31%, 22.05%, and 21.62%).

### 2.5. Phylogenetic Analysis

**Figure 7:**
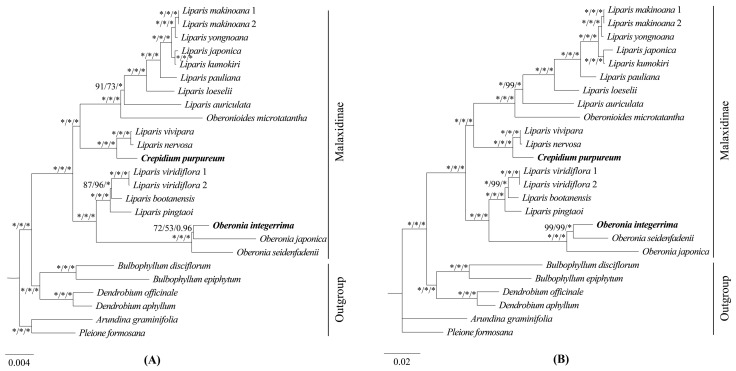
Phylogenetic tree of Malaxidinae obtained via maximum likelihood analysis based on 68 protein-coding regions (**A**) and 51 intergenic regions (**B**). Numbers near the nodes are bootstrap percentages and Bayesian posterior probabilities (BS_ML_, left; BS_MP_, middle; and PP, right). An asterisk (*) indicates the node has 100% bootstrap or 1.00 posterior probability.

**Figure 8:**
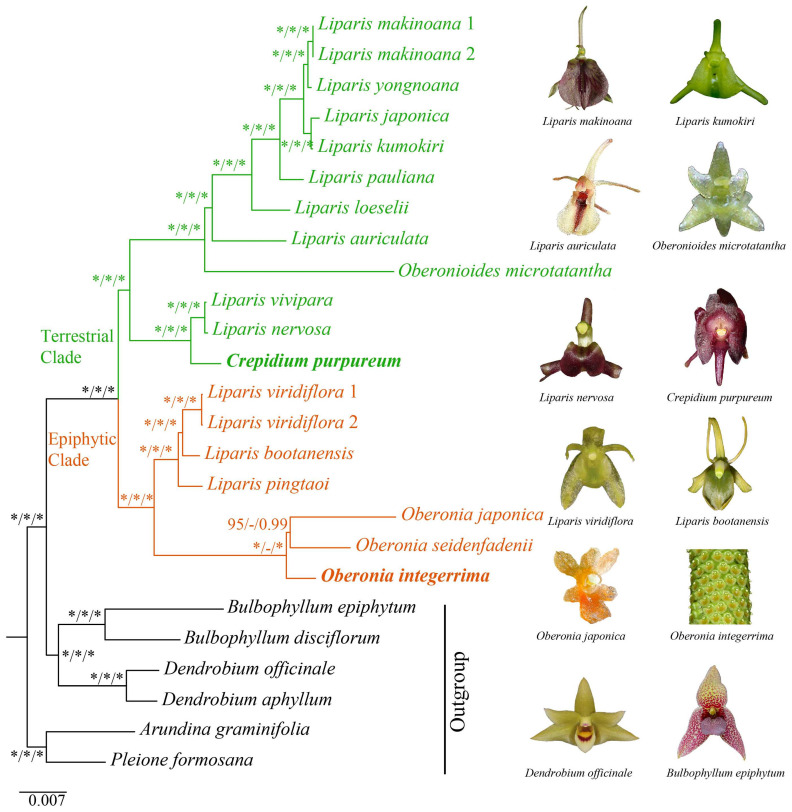
Phylogenetic tree of Malaxidinae obtained via maximum likelihood analysis based on whole plastome dataset. Numbers near the nodes are bootstrap percentages and Bayesian posterior probabilities (BS_ML_, left; BS_MP_, middle; and PP, right). An asterisk (*) indicates the node has 100% bootstrap or 1.00 posterior probability. Green represents the terrestrial clade, while orange represents the epiphytic clade. The species names in bold indicate those sequenced in this study.

Three matrices comprising 68 protein-coding genes (Figure 7A), 51 intergenic regions (Figure 7B), and complete plastomes (Figure 8) were employed to reconstruct the phylogenetic relationships of Malaxidinae. Three phylogenetic trees exhibited consistent topologies, except for the relationship among three species of *Oberonia* (Figure 7 and Figure 8). The bootstrap values for most nodes were ≥85 in maximum likelihood (ML) and maximum parsimony (MP) analyses and were ≥0.99 in Bayesian inference (BI) analysis. The phylogenetic relationships of the complete plastome (Figure 8) indicated that the newly sequenced *O. integerrima* forms a monophyletic group with two other species (*O. japonica* and *O. seidenfadenii*). This monophyletic group was sister to the clade containing *L. viridiflora* (Blume) Lindl., *L. bootanensis* Griff., and *L. pingtaoi* (G.D.Tang, X.Y.Zhuang & Z.J.Liu) J.M.H.Shaw. Additionally, the other newly sequenced species, *C. purpureum*, was strongly supported as sister to *L. vivipara* H.X.Huang, Z.J.Liu & M.H.Li, and *L. nervosa*. Furthermore, the *Oberonioides* was sister to the *Liparis* clade containing *L. auriculata* Blume ex Miq., *L. loeselii* (L.) Rich., *L. pauliana* Hand.-Mazz., *L. kumokiri* F.Maek., *L. japonica*, *L. yongnoana*, and *L. makinoana* Schltr. These results supported the assumption that *Liparis* was non-monophyletic.

**Figure 9:**
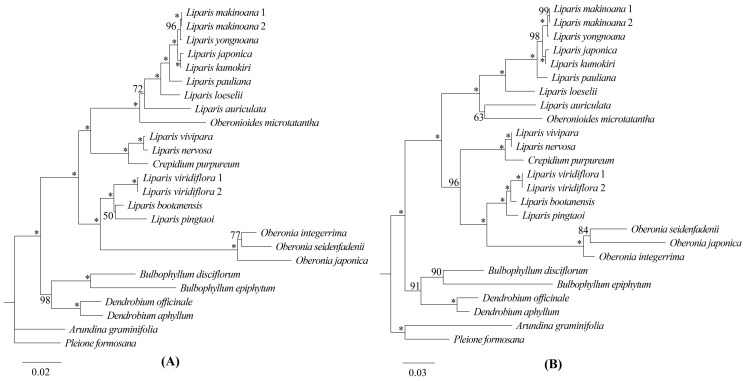
Phylogenetic tree of Malaxidinae obtained via maximum likelihood (ML) analysis based on the top-four protein-coding gene hotspots (*ycf1*, *matK*, *rps16*, and *rpl32*) (**A**) and the top-four intergenic region hotspots (*ccsA*-*ndhD*, *clpP*-*psbB*, *trnF^GAA^*-*ndhJ*, and *trnS^GCU^*-*trnG^UCC^*) (**B**). Numbers near the nodes are bootstrap percentages for ML analysis. An asterisk (*) indicates the node has 100% bootstrap probability.

Phylogenetic trees were constructed using the top four protein-coding gene hotspots (*ycf1*, *matK*, *rps16*, and *rpl32*) and the top four intergenic region hotspots (*ccsA*-*ndhD*, *clpP*-*psbB*, *trnF^GAA^*-*ndhJ*, and *trnS^GCU^*-*trnG^UCC^*), respectively, as selected based on the Pi value. Apart from the phylogenetic relationship within *Oberonia*, the topological structures of the two ML trees exhibited three distinctions compared with the reconstruction based on the complete plastomes. In the ML tree based on the top-four protein-coding gene hotspots, *L. bootanensi* was sister to *L. pingtaoi* with weak support and a short branch length, and this pair was sister to *L. viridiflora* (Figure 9A). In the ML tree using the top-four intergenic region hotspots, the clade, including *C. purpureum*, *L. vivipara*, and *L. nervosa*, had strong support as sister to the *Oberonia*, *L. pingtaoi*, *L. bootanensis*, and *L. viridiflora* clade (Figure 9B). *Oberonioides microtatantha* was sister to *L. auriculata*; moreover, this pair was sister to *L. loeselii* and its allies (Figure 9B). In 23 branch nodes, the bootstrap values for 20 nodes were ≥85 in the ML tree based on the combination of four protein-coding genes, whereas there were 21 nodes in the ML tree based on the combination of four intergenic regions. To further verify whether or not these molecular markers were applicable to other groups, 44 species of Epidendroideae were selected to reconstruct ML trees (Figure S1, Table S5). The results showed that most nodes had relatively strong support in the phylogenetic tree based on whole plastomes. However, significant topological conflicts were detected in the phylogenetic trees based on the four intergenic regions and four protein-coding genes with moderate to low support (Figure S1).

## 3. Discussion

### 3.1. Plastome Structure Conservation and Divergence

In this study, the sequenced plastome of *O. integerrima* and *C. purpureum* were 146,620 bp and 158,780 bp long, respectively, falling well within the typical range for autotrophic Orchidaceae plastomes, from 142,859 bp (*Schoenorchis seidenfadenii* Pradhan) to 178,131 bp (*Cypripedium formosanum* Hayata) [20,29]. The Malaxidinae species exhibited the typical quadripartite structure observed in angiosperm plastomes, containing LSC, SSC, and two IR copies. The size of the plastome ranged from 142,996 bp (*O. japonica*) to 158,787 bp (*L. nervosa*), the gene number range was 125 to 132, and all the species possessed 38 tRNA and 8 rRNA genes (Table S1). All of these characteristics were consistent with previously reported autotrophic Orchidaceae species [21,30,31]. The plastome size of *Oberonia* and *Oberonioides* (<14.7 kb) was smaller than that of *Liparis* and *Crepidium* (>15.0 kb) (Table S1). This discrepancy could be attributed to the loss or pseudogenization of the *ndh* genes, which was related to photosynthesis.

Previous studies have indicated that the loss or pseudogenization of *ndh* genes in the plastid genome was a common phenomenon in autotrophic Orchidaceae species [29,32,33]. Such patterns were generally associated with lifestyle, and they have occurred more frequently in the plastomes of epiphytic or lithophytic species, such as Aeridinae, *Bulbophyllum*, *Cymbidium*, and *Dendrobium* [20,30,34,35]. In this study, *Oberonia* and *Oberonioides* were epiphytic or lithophytic, suggesting a significant association between *ndh* gene loss or pseudogenization and epiphytic life forms. Notably, *L. japonica* and *L. yongnoana*, two terrestrial species, also lost *ndh* genes, and it is worth further investigation to better understand this phenomenon.

It is generally assumed that changes in IR regions are the main factors in the plastome size differences. Moreover, expansion and contraction at the IR/SC boundary are common evolutionary phenomena [36,37]. In this study, among the Malaxidinae plastomes, the JLA and JLB boundaries—located in the *psbA* and *rpl22* genes (except *L. japonica*), respectively—were highly conserved (Figure 3). Compared with *Liparis* and *Crepidium*, the loss of *ndhF* in *Oberonia* and *Oberonioides* resulted in a shift in the location of the JSB boundary to *trnN^GUU^*-*rps15*, leading to a contraction in the SSC region. Consequently, the plastomes of *Oberonia* and *Oberonioides* exhibited smaller genome sizes (Figure 3, Table S1).

Codons play a crucial role in genetic information transmission, mRNA stability, and accurately expressing protein functions [38]. Different species exhibited varying degrees of codon bias, influenced by selective pressures and translation efficiency during evolution [39,40]. In this study, the codon usage bias of Malaxidinae was similar overall. Leu presented the highest amino acid frequency and was closely associated with photosynthesis-related metabolism [41]. Conversely, Cys showed the lowest frequency, as excessive accumulation of Cys could lead to cellular oxidative damage and toxic responses [42]. For termination codons, UAA was preferred over UAG and UGA (Table S2), similar to other Orchidaceae species [40,43].

Previous studies have shown that the combined dataset of mutational hotspots regions was effective for identification analyses in certain groups [30,44]. In our study, the protein-coding genes were more conserved than the intergenic regions. Specifically, the *ycf1*, *matK*, *rps16*, and *rpl32* genes among the 68 protein-coding genes and the *ccsA*-*ndhD*, *clpP*-*psbB*, *trnF^GAA^*-*ndhJ*, and *trnS^GCU^*-*trnG^UCC^* regions among the 51 intergenic regions were mutation hotspots (Figure 6, Table S3). According to ML analyses of the two combinations, most branch nodes had strong support. Of the four protein-coding gene combinations, the phylogenetic relationship of the main clades of Malaxidinae showed good correspondence with the complete plastome-based phylogeny. However, the topology of the phylogenetic tree based on the combined intergenic regions indicated more significant inconsistencies. Therefore, this combination, containing *ycf1*, *matK*, *rps16*, and *rpl32*, is a more ideal molecular marker for phylogenetic and species identification analyses within the genus of Malaxidinae. To further explore the applicability of these markers across different taxonomic groups, phylogenetic relationships were constructed using ML analysis based on whole plastomes, four protein-coding genes, and four intergenic regions. The results revealed significant incongruence between the phylogenetic trees inferred from the four protein-coding genes and four intergenic regions compared to those based on whole plastomes (Figure S1). These findings suggest that these molecular markers appear to be specific to Malaxidinae and may not be suitable for other taxonomic groups.

### 3.2. Phylogenetic Relationships

The molecular-based phylogenetic relationships of Malaxidinae were inconsistent with the traditional morphological classification. Cameron et al. [4] constructed phylogenetic relationships for this group and combined them with lifestyle to support dividing Malaxidinae into two major clades: terrestrial clade and epiphytic clade. Likewise, Margońska et al. [10], Salazar et al. [13], Li et al. [16], and Kumar et al. [17] expanded the sampling to include additional genera. Their molecular phylogenetic reconstructions showed that *Liparis*, *Malaxis*, *Oberonia*, and *Crepidium* were embedded within each other, and multiple genera were confirmed as polyphyletic. However, these phylogenetic relationships showed unstable topologies with moderate-to-weak support. Some authors have proposed excluding the non-monophyletic taxa of *Liparis* and *Malaxis* into several small genera to ensure the monophyly of this group. These unreliable topologies might reflect unreliable phylogenetic relationships, resulting in incorrect taxonomic revision. In addition, incongruence among the ML, MP, and BI analyses was detected in the phylogenetic tree based on the whole plastome dataset. This phenomenon might be attributable to systematic errors or biological factors. Further studies using more phylogenetic methods and broader material sampling are needed.

Our phylogenetic study utilized high-throughput sequencing to obtain complete plastomes. The phylogenetic relationships were determined using ML, MP, and BI methods with complete plastomes, protein-coding genes, and intergenic regions. The results showed that the topology structures were almost the same within the ML, MP, and BI analyses; furthermore, most branch nodes were strongly supported (BS ≥ 85, PP ≥ 0.99) in three phylogenetic trees (Figure 7 and Figure 8). This finding provides robust evidence supporting the utility of plastid genomes as molecular markers and provides a practical approach for phylogenetics in this subtribe.

Considering the lifestyle of these species (Table S4), our phylogenetic trees demonstrated that Malaxidinae was clustered into two major clades, an epiphytic clade and a terrestrial clade (Figure 8), which was strongly congruent with previous results [4,10,17]. In three phylogenetic analyses, the relationships among *O. integerrima*, *O. japonica*, and *O. seidenfadenii* varied slightly with moderate support values (Figure 7 and Figure 8). Our analysis confirmed that *Crepidium*, *Oberonia*, and *Oberonioides* were nested within *Liparis*, indicating that *Liparis* was non-monophyletic (Figure 8). These results suggest the need to redefine the relationships among these genera, which requires broader sampling to extend the phylogenomic datasets. Overall, our phylogenomic analyses significantly improved the stability of the topology structure and support values of the plastid phylogeny than previous studies.

## 4. Materials and Methods

### 4.1. Sample Preparation, Sequencing, and Data Acquisition

In this study, *Oberonia integerrima* and *Crepidium purpureum* were collected from Puer, Yunnan province, and Zhangzhou, Fujian province, China, respectively. Young and healthy leaves were sampled for sequencing on 25 August 2021. Voucher specimens were deposited in the herbarium of the Forestry College of Fujian Agriculture and Forestry University (FJFC) under specimen codes MHLi or119 and MHLi or110. Additionally, 17 published sequences (15 species) of Malaxidinae and 6 sequences as an outgroup were downloaded from the GenBank database (Table S4).

The total DNA was extracted from fresh leaves using the Plant Mini Kit (Qiagen, Redwood City, CA, USA) based on the manufacturer’s protocol. The DNA integrity was evaluated via electrophoresis on 1% agarose gels, and DNA sample preparations with over 1 μg of DNA per sample were selected. The total DNA samples were randomly sheared, and libraries for paired-end 150 bp sequencing were conducted using an Illumina HiSeq 4000 platform (Illumina, San Diego, CA, USA) to generate approximately 20 Gb of raw data per sample. The quality of the newly generated sequencing data was assessed using the FastQC software (http://www.bioinformatics.babraham.ac.uk/projects/fastqc (accessed on 12 March 2022)) [45].

### 4.2. Plastome DNA Assembly, Annotation, and Comparison

The raw data were filtered for low-quality sequencing reads to obtain plastid-like reads. The whole plastome sequences were assembled using the Perl script get_organelle_from_reads.py in GetOrganelle pipeline v1.7.1 [46] with *k*-mer ranges of 21, 45, 65, 85, and 105 for the plastomes. The published *L. bootanensis* plastome (MN627759), which was downloaded from GenBank on 6 May 2022, had longer assembly length and complete genes and was closely related to two newly sequenced species. It was used as a reference for assembling and annotating the plastome. The “fastg” and “cvs” files were imported to Bandage [47]; then, the low-quality fragments were filtered and edited to obtain the circular plastomes. With the *L. bootanensis* reference, the assembled plastomes were annotated by the Perl script PGA.pl in Plastid Genome Annotator (PGA) [48] with default parameters, including -i, 1000, -p 40 -q 0.5,2. The results were corrected and adjusted using the Dual Organellar Geno Me Annotator (DOGMA) [49]. For high-quality annotation plastomes, we used Geneious 11.1.5 (https://www.geneious.com (accessed on 1 August 2022)) to align with the *L. bootanensis* reference, manually adjusting the position of the initiation and termination codons and examining the loss or pseudogenization of each gene, particularly the *ndh* genes. The sequencing data were submitted to GenBank. The published sequences in GenBank were re-annotated by the same steps. The 19 visible circle annotation results were drawn with the online tool OGDRAW (http://ogdraw.mpimp-golm.mpg.de/ (accessed on 9 October 2022)) [50].

### 4.3. Plastome Features Analysis

Annotated plastome genome information for 19 Malaxidinae species and 6 species of outgroup was collected, including gene length; GC content; size of IR, LSC, and SSC; the number of genes; and *ndh* genes loss/pseudogenization. Using the *L. bootanensis* reference, the complete plastomes of Malaxidinae were aligned with mVISTA in Shuffle-LAGAN mode [51] to assess the variability of the sequences. The boundary regions of LSC/IRb/SSC/IRa were analyzed with IRscope (https://irscope.shinyapps.io/irapp (accessed on 20 October 2022)) [52]. The rearrangement and inversion of the plastomes were detected and graphed with Mauve [53].

### 4.4. Codon Usage and Nucleotide Mutation Hotspots Analysis

All protein-coding genes and intergenic regions of the 25 samples (23 species) were extracted with the Extract GenBank file program in PhyloSuite v1.2.1 [54] using codon alignment mode. Then, we deleted one copy gene of the IR and *ndh* genes and retained the matrix with more than 20 class groups, resulting in a dataset of 68 protein-coding genes and 51 intergenic regions. These genes and regions were aligned individually using MAFFT v7 [55] with default parameters and concatenated using Phylosuite v1.2.1. The variable site (V) and parsimony information site (Pi) were counted using MAFFT v7 to determine highly variable regions of Malaxidinae. The RSCU of 19 Malaxidinae plastomes and codon frequencies were calculated using DAMBE [56]. To reduce sampling errors, we excluded protein-coding genes shorter than 300 bp from the codon usage analysis. This step was essential, as short protein-coding genes can cause inaccuracies in codon usage estimates. Finally, a heatmap was generated using Tbtools [57].

### 4.5. Phylogenetic Analysis

The phylogenetic relationships were analyzed with three alignment matrices: complete plastomes; protein-coding genes; and intergenic regions using ML, MP, and BI. Using the Convert Sequence Format script in Phylosuite v1.2.1 [54], the three alignment matrices were converted into “phy” and “nex” files for further phylogenetic analysis. Under the GTRGAMMA model of evolution [58], ML analyses were conducted in the CIPRES Science Gateway (RAxML-HPC2 on XSEDE 8.2.12) [59]. Bootstrap iterations were performed with 1000 bootstrap replicates using heuristic searches [60], with the other parameters set to default values. For MP analysis, 1000 random addition sequence replicates were used with a heuristic search about 1000 tree-bisection-reconnection (TBR) branch-swapping, treating all characters as unordered and of equal weight [61]. BI analysis was performed using MrBayes v.3.2.6 with the GTR + I + Γ substitution model [62]. To ensure the reliability of results, the Markov chain Monte Carlo (MCMC) algorithm was run for 10,000,000 generations, with one tree sampled every 100 generations. The first 25% of the resulting trees were discarded as burn-in to construct majority-rule consensus trees and estimate posterior probabilities (PP).

Based on nucleotide mutation hotspot analysis, the top four mutational hotspots from the protein-coding genes and intergenic regions were identified separately based on Pi value. Two sets of these hotspots were concatenated using Phylosuite v1.2.1 [54]. The phylogenetic relationships were constructed with these two alignment matrices using ML; the detailed steps are referred to in the preceding text. Then, the ideal molecular marks were identified by the comparison with the complete plastome-based phylogeny. A total of 44 species were selected to construct the phylogenetic tree within the subfamily Epidendroideae.

## 5. Conclusions

In this study, two plastomes of *O. integerrima* and *C. purpureum* were newly sequenced. Except for an inversion with the *rpl33*-*rps3* region in *Oberonioides microtatantha*, the Malaxidinae plastome structures were highly conserved. The plastome size of *Oberonia* and *Oberonioides* was smaller than that of *Liparis* and *Crepidium*, due to the loss or pseudogenization of *ndh* genes and the contraction of the SSC region. Four protein-coding genes (*ycf1*, *matK*, *rps16*, and *rpl32*) and four intergenic spacers (*ccsA*-*ndhD*, *clpP*-*psbB*, *trnF^GAA^*-*ndhJ*, and *trnS^GCU^*-*trnG^UCC^*) were identified as mutation hotspots. The combination of *ycf1*, *matK*, *rps16*, and *rpl32* could be used in the phylogeny and identification of the genus level within Malaxidinae. The phylogenomic analysis strongly supported that Malaxidinae was clustered into two clades, namely, the epiphytic clade and the terrestrial clade. The *Liparis* genus was shown to be non-monophyletic.

## Data Availability

All the data are provided within this manuscript and Supplementary Materials.

## Supplementary Materials

The supporting information can be downloaded at: https://www.mdpi.com/article/10.3390/ijms252011181/s1.
