# Supplementary material for: Comparative Phylogenomic Study of Malaxidinae (Orchidaceae) Sheds Light on Plastome Evolution and Gene Divergence
Source: Int J Mol Sci. 2024 Oct 17;25(20):11181. doi: 10.3390/ijms252011181 (PMC11508673; doi:10.3390/ijms252011181)
Supplement: Supplementary file 1 [file ijms-25-11181-s001.zip › Supplementary Figure S1.pdf]

Supplementary Figure

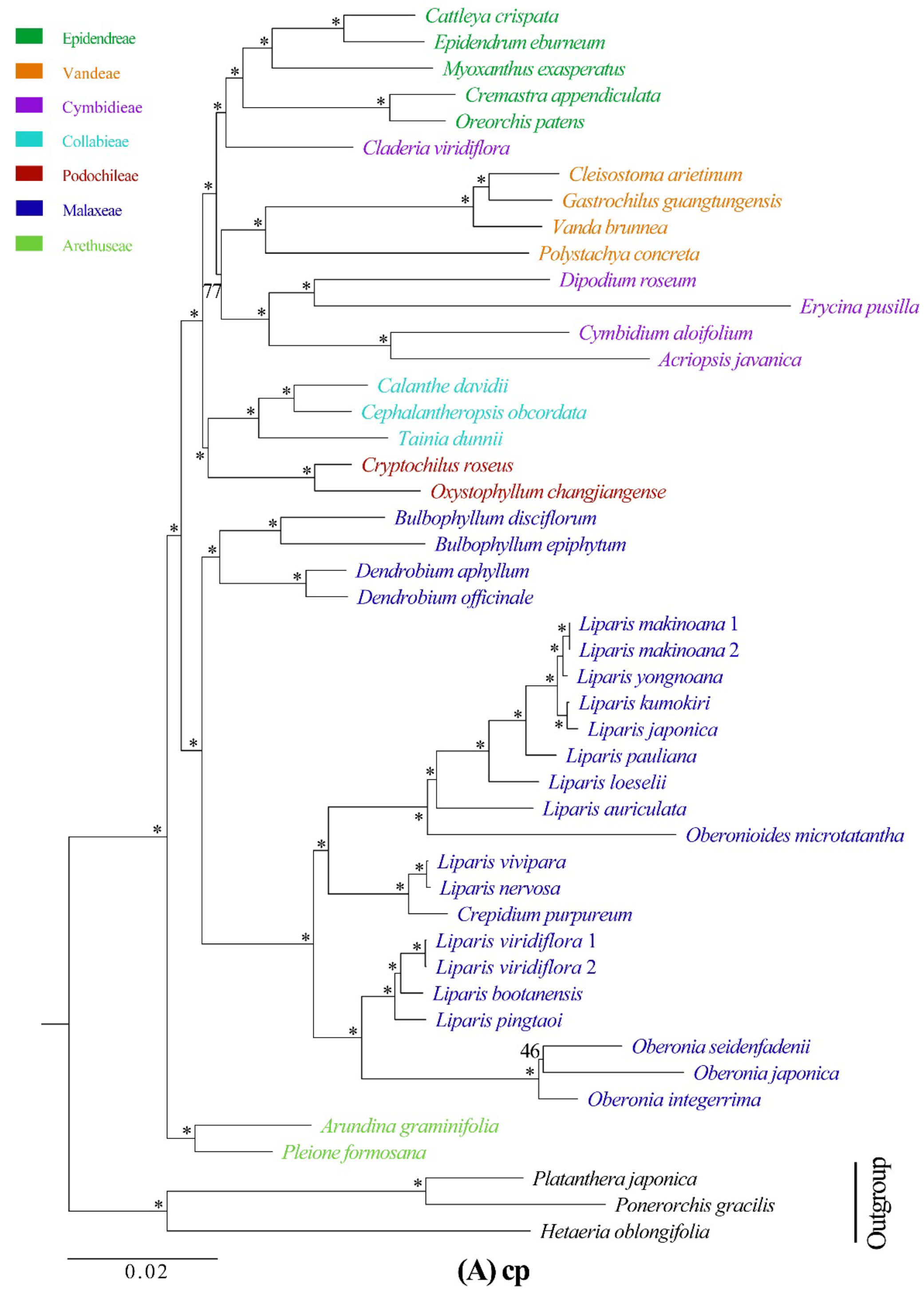

(A) cp

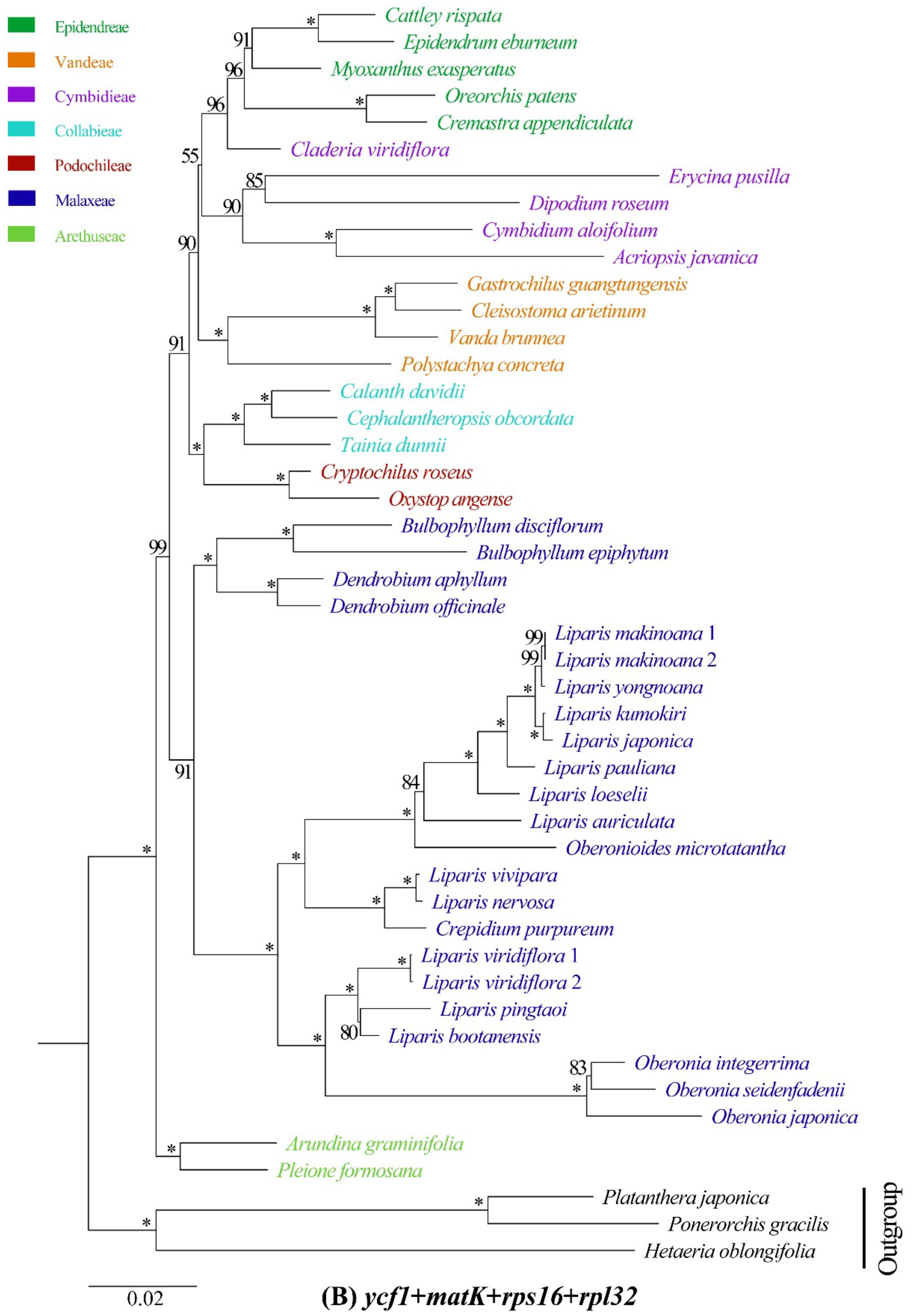

(B) *ycf1+matK+rps16+rpl32*

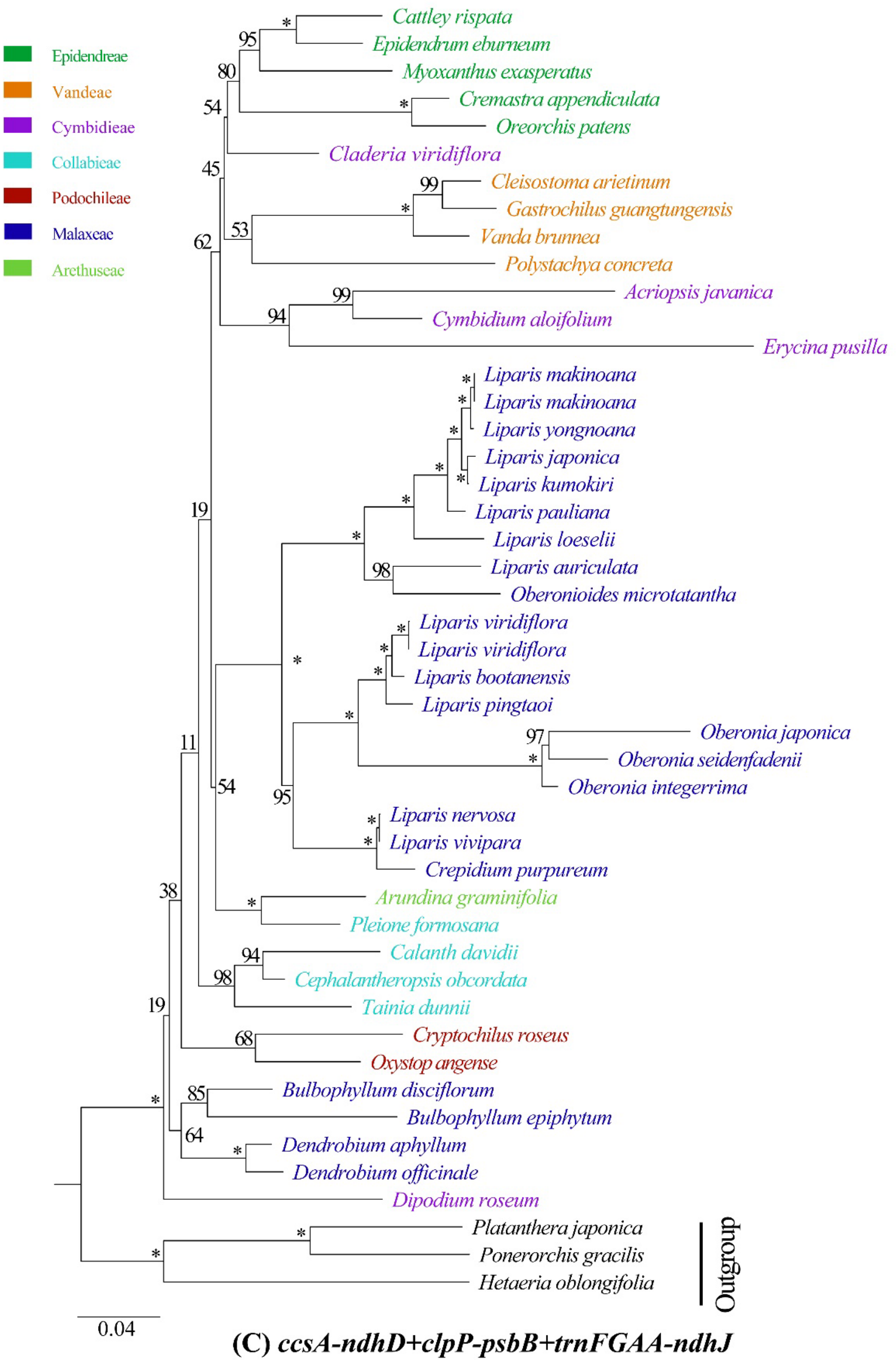

(C) *ccsA-ndhD+clpP-psbB+trnFGAA-ndhJ +trnSGCU-trnGUCC*

Figure S1. Phylogenetic tree of Epidendroideae obtained viamaximum likelihood (ML) analysis based on the whole plastome (A), the top-four protein-coding genes hotspots (*ycf1*, *matK*, *rps16*, and *rpl32*) (B) and the top-four intergenic regions hotspots (*ccsA-ndhD*, *clpP-psbB*, *trnF*^{GAA}*-ndhJ*, and *trnS*^{GCU}*-trnG*^{UCC}) (C). Numbers near the nodes are bootstrap percentages for ML analysis. An asterisk (*) indicates the node has 100% bootstrap probability.
